# Burden and Patterns of Multidrug-Resistant Tuberculosis in Pediatric and Adolescent Patients at a Tertiary Care Hospital in Pakistan

**DOI:** 10.7759/cureus.63246

**Published:** 2024-06-26

**Authors:** Ghulam Mustafa, Feras Almarshad

**Affiliations:** 1 Department of Pediatric Medicine, College of Medicine, Shaqra University, Shaqra, SAU; 2 Department of Pediatrics, Nishtar Medical University and Hospital, Multan, Multan, PAK; 3 Department of Internal Medicine, College of Medicine, Shaqra University, Shaqra, SAU

**Keywords:** pakistan, multidrug-resistant tb (mdr tb), adolescent tb, malnutrition, epidemiology, drug resistance, pediatric tb

## Abstract

Background

Multidrug-resistant tuberculosis (MDR-TB) presents a significant global health challenge, particularly in developing countries. This study focuses on the burden and pattern of pediatric and adolescent MDR-TB in a tertiary care hospital setting.

Aims/objectives

The main objective is to evaluate MDR-TB’s prevalence and resistance patterns among pediatric and adolescent patients, highlighting critical demographic factors and resistance trends.

Materials and methods

The study utilized a prospective analytical design in two tertiary care facilities, focusing on children aged four months to 18 years diagnosed with extra-pulmonary tuberculosis. Data on demographic profiles, clinical outcomes, and drug resistance patterns were collected and analyzed using the Statistical Package for the Social Sciences (IBM SPSS Statistics for Windows, IBM Corp., Version 27.0, Armonk, NY).

Results

Out of 99 enrolled participants, 63 (63.64%) met the inclusion criteria. The mean age was 70.22±48.90 months. A significant proportion, 60 (95.2%) of the cases, originated from Punjab. Notably, 10 (15.9%) of the cultures demonstrated MDR, with specific resistance observed to isoniazid (INH) in 10 (15.9%) cases, rifampicin (RIF) in 11 (17.5%) cases, and pyrazinamide (PZA) in seven (11.1%) cases. The study also recorded a high prevalence of tuberculous meningitis, affecting 52 (82.5%) participants, and malnutrition, affecting 49 (77.8%).

Conclusions

MDR-TB in 10 (15.9%) of the study children and adolescents presenting in Pakistan’s specialized health centres is a notable burden. This points to a need for better diagnostic methods and treatment plans for pediatric patients. Implementing advanced diagnostics and personalized therapies is crucial for managing MDR-TB in susceptible demographics. Our findings emphasize the importance of updating treatment protocols to tackle the impacts of MDR-TB and its evolving resistance.

## Introduction

Multidrug-resistant tuberculosis (MDR-TB) is a severe and widespread issue, particularly prevalent in many developing countries due to suboptimal TB treatment practices globally [1]. Observations indicate a 1-2% global incidence of MDR-TB among all TB cases, which disproportionately impacts less developed nations and impedes effective TB control measures [2]. MDR-TB arises from the mismanagement of treatable TB cases, leading to drug resistance and incomplete treatment regimens [1,2]. The challenges associated with MDR-TB include the need for extensive medication regimens, which can have significant adverse effects, an elevated mortality rate compared to non-resistant TB, and substantial investments of time and financial resources required for accurate diagnosis [2].

*Mycobacterium tuberculosis* (M. tb) is classified as MDR when it exhibits resistance to both isoniazid (INH) and rifampicin (RIF), which are the most effective and least harmful first-line anti-TB medications, with or without resistance to other drugs. This resistance is due to genetic mutations [2,3]. These drugs are crucial for preventing, treating, and curbing the spread of TB, and they form the cornerstone of TB control strategies. While resistance to either INH or RIF can be managed individually, simultaneous resistance to both necessitates the use of second-line drugs, leading to treatments that are longer, more expensive, and associated with significant adverse effects [4,5]. Since 1984, fluoroquinolones, a class of broad-spectrum antibiotics, have been central in managing MDR-TB due to their effectiveness [2].

In 2019, there were approximately 464,000 cases of RIF-resistant TB globally, with 78% (362,320) classified as MDR-TB cases [6]. By 2021, the number of reported MDR-TB cases had increased to an estimated 450,000, representing a 3.1% rise from the previous year [6]. Additionally, about 25% of TB-related deaths are attributed to antimicrobial drug resistance [2]. Research has shown that MDR-TB can lead to significant and long-term consequences, impacting physical health, mental well-being, and financial stability [7]. Between 2000 and 2007, Turkey experienced a 29% resistance rate to any TB drug, with MDR-TB accounting for 4.5% of those cases [8]. MDR-TB prevalence was also notable in the UAE (9.2%), Kuwait (5.9%), Saudi Arabia (4.3%), Brazil (3.5%), and Germany (2.2%) [9,10]. Additionally, a 3.4% prevalence of MDR-TB was observed in newly diagnosed cases [2,6,11].

Pakistan grapples with approximately 510,000 new TB cases and about 15,000 new cases of drug-resistant TB each year, ranking it as the fifth highest among countries burdened by TB globally and accounting for 61% of the TB burden in the WHO Eastern Mediterranean Region [12]. Additionally, Pakistan is estimated to be fourth worldwide in terms of the prevalence of MDR-TB [12]. Patients with MDR-TB in Pakistan show a lower recovery rate compared to those with drug-sensitive TB [13].

This study aims to illuminate the occurrence and patterns of MDR-TB at a tertiary care hospital, specifically focusing on pediatric and adolescent patients undergoing treatment in a developing country.

## Materials and methods

We utilized a convenient sampling technique and implemented an open, prospective analytical design for our study, which was conducted in the Pediatric Departments of two tertiary care institutions: Nishtar Medical University and the Institute of Child Health and Children Complex, located in Multan.

To determine the sample size, we utilized a prevalence-based formula. Assuming a prevalence rate of 10% for extra-pulmonary tuberculosis (EPTB) and aiming for a margin of error of 5%, we calculated the sample size necessary to achieve a 90% confidence interval. The Z-value corresponding to a 90% confidence level is 1.645. Utilizing the formula n=(Z^2^×P(1−P))/E^2^, where P is the estimated prevalence (10%), E is the margin of error (5%), and Z is the Z-score associated with the desired confidence level, we determined that a sample size of approximately 97 participants is required.

The study included inpatient children and adolescents aged four months to 18 years diagnosed with EPTB, scoring 7 or higher on a diagnostic scale devised by the Pakistan Pediatric Association to identify TB and its extra-pulmonary manifestations. We excluded individuals younger than four months or older than 18 years, those with diagnostic scores below 7, patients diagnosed with pulmonary TB (except miliary TB), and children with storage or metabolic disorders, primary immunodeficiency, malignancies, encephalitis, or bacterial (but not tuberculous) meningitis. Data collection was thorough, utilizing a detailed proforma completed by a trained doctor, capturing biodata, medical history, physical examination results, complete blood counts, erythrocyte sedimentation rate (ESR), chest X-ray, tuberculin tests (TT), and diagnostic Bacillus Calmette-Guérin (BCG), along with imaging studies like computed tomography (CT) scans or magnetic resonance imaging (MRI).

Culture and drug susceptibility tests for M. tb isolates, primarily sourced from cerebrospinal fluid (CSF), were conducted using the Löwenstein-Jensen (LJ) medium at the Tuberculosis Research Center, Mayo Hospital, Lahore. Other sources of isolates included lymph nodes, pleural fluids, joint aspirates, peritoneal fluids, and urine. Drug susceptibility was tested using the standard proportion method at specific drug concentrations: RIF 40.0 μg/mL, INH 0.2 μg/mL, streptomycin (STM) 4.0 μg/mL, ethambutol (EMB) 2.0 μg/mL, and pyrazinamide (PZA) 100.0 μg/mL. For PZA, the medium was acidified to a pH of 5.5. Two bacterial suspension dilutions were inoculated on drug-containing and drug-free media for control. Quality control was maintained using a drug-susceptible M. tb strain. We incubated the tubes at 37°C for a minimum of four weeks, after which we compared the colony growth on the drug-containing media to the growth on drug-free media to determine resistance. Resistance was defined as more than 1% growth for INH, EMB, and RIF and 10% for STM and PZA, compared to the control. This procedure follows standard guidelines provided by the WHO and the Clinical and Laboratory Standards Institute (CLSI). The analysis also considered the patients’ treatment history, distinguishing between primary resistance in treatment-naïve patients and acquired resistance in those previously treated.

The Ethical Review Committee at Nishtar Medical College and Hospital, Multan approved this study, reference number 7532/44/NMC, Multan. The investigators adhered strictly to all ethical and consent guidelines and regulations throughout this study.

For our statistical analyses of MDR patterns among the specimens and the distribution of EPTB types, we utilized the Statistical Package for the Social Sciences (IBM SPSS Statistics for Windows, IBM Corp., Version 27.0, Armonk, NY). Categorical data were summarized using frequencies and percentages, while quantitative data were described with means and standard deviations.

## Results

A total of 99 infants, children, and adolescents were enrolled in the study. Of these, 63 (63.64%) were included in the final analysis based on the diagnostic criteria, the availability of culture results, and the completeness of the data. The mean age of the children was 70.22±48.90 months. The median age, mode, and age range were 66.67 months, 72 months, and four to 180 months, respectively. Most children, 59 (79.3%), were between 1 and 12 years old. The male-to-female ratio was relatively balanced, with 34 males (56%) and 29 females (46%). A significant majority of the participants, 52 (82.5%), were diagnosed with tuberculosis meningitis (TBM) while 49 (77.8%) suffered from malnutrition. A history of contact with TB patients was noted in 25 (39.7%) of the children, and only seven (11.1%) had previously received anti-tuberculosis treatment (ATT) for various durations, as detailed in Table 1. This table also underscores a comprehensive analysis of the demographic traits of the study sample and their association with MDR-TB. The overwhelming majority of the cases, 60 (95.2%), came from Punjab, pointing out the regional prevalence and potentially identifying areas where public health interventions may be most necessary.

**Table 1:** Demographic characteristics and their relationship with multidrug-resistant tuberculosis (MDR-TB) TBM: tuberculosis meningitis; TB: tuberculosis; ATT: anti-tuberculosis treatment

Characteristics	n (%)
Age (months) mean ± SD	70.22±48.90
Age Groups	
<1 year	9 (14.3)
1-5 year	21 (33.3)
5-12 year	29 (46.0)
>12 year	4 (6.3)
Gender	
Male	34 (56)
Female	29 (46)
Province	
Baluchistan	1 (1.6)
KPK	2 (3.2)
Punjab	60 (95.2)
Malnutrition	
Grade 1	2 (3.2)
Grade 2	6 (9.5)
Grade 3	41 (65.1)
No malnutrition	14 (22.2)
History of Measles in Last Two Months	1 (1.6)
History of Whooping Cough in Last Two Months	2 (3.2)
History of Contact With TB Patient	25 (39.7)
Diagnostic Variation	
TBM	52 (82.5)
Lymph node TB	4 (6.3)
Urinary TB	1 (1.6)
Pleural TB	3 (4.8)
TB Abdomen	2 (3.2)
Miliary TB	1 (1.6)
Previous h/o treatment with ATT	7 (11.1)
Duration of ATT taken (in months)	
No ATT taken	56 (88.9)
2	1 (1.6)
4	2 (3.2)
6	2 (3.2)
9	2 (3.2)
Acid-Fast Bacilli Positive	11 (17.5)

Table 2 describes the drug sensitivity profiles of M. tb isolates against an array of first-line drugs, focusing on the proportion of isolates that have demonstrated resistance or sensitivity to each tested drug. Notably, resistance was noted in 10 (15.9%) of the isolates for INH, 11 (17.5%) for RIF, and seven (11.1%) for PZA, while sensitivity was observed in five (7.9%), four (6.3%), and eight (12.7%) of the samples, respectively. Additionally, 10 (15.9%) samples demonstrated MDR. These figures underscore the challenges in managing TB, highlighting prevalent resistance patterns and underscoring the necessity for healthcare professionals to customize treatment strategies. It is important to note that in 48 (76.2%) of the samples, no growth was observed, which is critical for assessing the effectiveness of existing drug regimens. The detailed resistance patterns to other first-line ATT drugs and combinations are also presented in Table 2, providing a comprehensive overview of the drug-resistance dynamics observed within pediatric demographics.

**Table 2:** Mycobacterium tuberculosis isolates and their sensitivities to various anti-tuberculosis drugs INH: isoniazid

Anti-tuberculosis Drugs and Their Sensitivity Pattern	n (%)
No Growth	48 (76.2)
INH	
Resistant	10 (15.9)
Sensitive	5 (7.9)
Rifampicin	
Resistant	11 (17.5)
Sensitive	4 (6.3)
Pyrazinamide	
Resistant	7 (11.1)
Sensitive	8 (12.7)
Ethambutol	
Resistant	5 (7.9)
Sensitive	10 (15.9)
Streptomycin	
Resistant	5 (7.9)
Sensitive	10 (15.9)
INH and Rifampicin	
Resistant	10 (15.9)
INH and Pyrazinamide	
Resistant	6 (9.5)
INH and Ethambutol	
Resistant	4 (6.3)
INH and Streptomycin	
Resistant	3 (4.8)
Rifampicin and Pyrazinamide	
Resistant	6 (9.5)
Rifampicin and Ethambutol	
Resistant	5 (7.9)
Rifampicin and Streptomycin	
Resistant	4 (6.3)
Pyrazinamide and Ethambutol	
Resistant	3 (4.8)
Pyrazinamide and Streptomycin	
Resistant	3 (4.8)
Ethambutol and Streptomycin	
Resistant	4 (6.3)
INH, Rifampicin and Pyrazinamide	
Resistant	6 (9.5)

**Figure 1:**
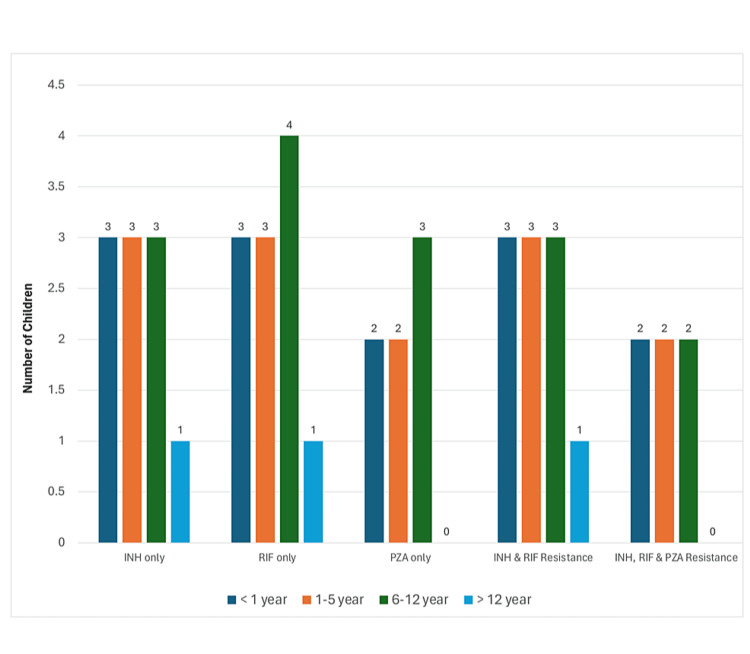
ATT drug resistance with age groups ATT: anti-tuberculosis treatment; INH: isoniazid; RIF: rifampicin; PZA: pyrazinamide

Figure 1 depicts the resistance patterns of drugs used in ATT among various age groups. The visual representation complements the tabulated data by illustrating the variation in drug resistance across different age groups. The MDR-TB affects children under 12 years preferentially. This is crucial for identifying the age groups that are most vulnerable and may need more intensive management or innovative treatment methods.

## Discussion

There have been various studies from Pakistan focusing on MDR-TB. However, ours is the first to focus on pediatric and adolescent EPTB. The findings of our study underscore a notable burden of MDR-TB among infants, children, and adolescents in a tertiary care hospital setting in a developing country. With 63.64% of enrolled participants (63 out of 99) meeting the inclusion criteria for the final analysis, the study reflects a considerable sample that elucidates the pattern of TB resistance within a pediatric demographic.

Our study has provided significant insights into the burden and pattern of pediatric and adolescent MDR-TB. The observed resistance patterns show alarming levels of resistance to critical first-line drugs, including INH at 10 (15.9%), RIF at 11 (17.5%), and PZA at 7 (11.1%). Furthermore, 10 (15.9%) samples were identified as MDR strains, underscoring the severe penetration of drug-resistant TB into pediatric populations. The fact that 10 (15.9%) samples exhibited resistance to both INH and RIF highlights a concerning trend that aligns with global data reported in recent studies.

A systematic review and meta-analysis of the global prevalence of drug-resistant TB revealed that the global pooled prevalence of MDR-TB is 11.6% (95% CI: 9.1-14.5%) [2]. Local studies have reported similar figures in the pediatric population, with one tertiary care hospital in Lahore, Pakistan, documenting MDR-TB in 26 (12.9%) of cases [14]. Research from the nodal drug-resistant TB centre at King George Medical University in India showed varying prevalences over four consecutive years: 17.4% in 2018, 15.1% in 2019, 18.4% in 2020, and 20.3% in 2021 [15]. A comprehensive national study in Germany found that four (22.2%) of the German-born population had MDR-TB. This study also noted that the prevalence of MDR-TB was four (22.2%) among children born in the Commonwealth of Independent States (including Azerbaijan, Moldova, and Russia), three (16.7%) in children born in Europe, six (33.3%) in those born in Africa, and one (11.1%) in children from Australasia (Iraq, Syria, Afghanistan, Norfolk Island) [10]. Another local study identified 35 (36.5%) samples as MDR-TB [16], while a nationwide study reported MDR-TB in 85 (34.85%) of isolates [17]. These findings highlight potential local challenges in drug management or adherence issues in pediatric TB treatments. Occasionally, pediatric tertiary care centres report as low as four (3.77%) MDR-TB cases, reflecting the diverse populations they serve [18].

The mean age of the children in our study, slightly over five years, along with the predominance of participants aged between 1 and 12 years, 50 (79.3%), underscores the susceptibility of this young demographic to TB and its drug-resistant forms. Regarding demographic distribution, our findings are consistent with another study, which reported that children under 13 comprise nine (34.6%) of all MDR-TB cases [14]. This correlation underscores the widespread impact of TB on younger populations globally.

Nearly 40% of the children in our study had a history of contact with TB patients, highlighting the critical role of transmission within family or community settings. This emphasizes the need for robust contact tracing and preventive therapy to control the spread of TB. The contact rate in our study was 25 (39.7%), significantly lower than the 16 (66.7%) reported in the *European Journal of Pediatrics* [10], indicating regional variations in transmission dynamics and the effectiveness of public health interventions.

The gender distribution in our study revealed a nearly equal sex ratio, suggesting that MDR-TB affects both males and females similarly, thereby discounting gender as a significant risk factor. This finding is consistent with some reports but contrasts with others that suggest a gender predisposition to TB susceptibility [16]. The high concentration of cases from Punjab, 60 (95.2%), is primarily due to the location of our tertiary care centre, which facilitates easier access for patients in this region.

We found that 52 (82.5%) of the children in the study group had TBM, and 49 (77.8%) were malnourished. It underscores the significant interaction between nutritional status and the severity of TB. This correlation supports studies that identify malnutrition as a critical risk factor for TB, especially for severe forms such as TBM [19]. These results emphasize the need for comprehensive healthcare approaches that integrate nutritional support to mitigate the impact of TB.

Our study indicates a notably high percentage of samples, 48 (76.2%), showing no growth, which could highlight difficulties in culturing samples from pediatric patients or suggest limitations in the sensitivity of culture methods for this age group. This trend aligns with findings from other research, suggesting it is a common issue encountered in pediatric TB studies [19,20].

The implications of this study are manifold. The high burden of MDR-TB among children, particularly those with malnutrition and TBM, calls for urgent enhancements in pediatric TB diagnosis, treatment, and nutritional support frameworks. Our results also highlight the critical need for developing more effective and child-friendly TB diagnostic and therapeutic tools. Additionally, the significant history of contact with known TB cases among the study participants necessitates intensified contact tracing and preventive treatment endeavours, particularly in TB-endemic regions like Punjab.

Regarding limitations, our focus on a tertiary care centre may not fully capture the community-based prevalence and burden of pediatric and adolescent MDR-TB, potentially biasing our results towards more severe cases. Another limitation is that it is not known whether all patients were regular on their treatment before and during the study period. This lack of information on treatment adherence may affect the study outcomes. Nonetheless, our study sheds light on the critical issues surrounding the management and control of pediatric and adolescent MDR-TB in developing countries.

Future research should aim at longitudinal and community-based studies to explore the epidemiology of pediatric TB more comprehensively. Investigations into the bio-social determinants of TB transmission, the development of more sensitive and specific pediatric TB diagnostic tools, and the formulation of child-friendly drug regimens for both TB treatment and MDR-TB are urgently needed.

## Conclusions

The results of our study provide clear evidence of the high occurrence and intricate patterns of MDR-TB in children and teenagers receiving medical treatment at advanced healthcare facilities in Pakistan. More precisely, our analysis revealed significant levels of resistance to INH in 10 (15.9%), RIF in 11 (17.5%), and PZA in seven (11.1%). Furthermore, 10 (15.9%) samples demonstrated resistance to INH and RIF (MDR), highlighting the urgent need for specific improvements in diagnostic approaches and treatment plans designed for young patients. Healthcare systems should look into incorporating advanced diagnostic tools and formulating personalized treatment protocols to better manage MDR-TB in vulnerable groups. Moreover, our findings stress the necessity for ongoing adjustments to treatment methodologies to lessen the profound effects of this serious disease on children and adolescents and to address the changing resistance patterns effectively.
